# The impact of treatment delivery format on response to cognitive behaviour therapy for preadolescent children with anxiety disorders

**DOI:** 10.1111/jcpp.12872

**Published:** 2018-03-09

**Authors:** Anna McKinnon, Robert Keers, Jonathan R.I. Coleman, Kathryn J. Lester, Susanna Roberts, Kristian Arendt, Susan M. Bögels, Peter Cooper, Cathy Creswell, Catharina A. Hartman, Krister W. Fjermestad, Tina In‐Albon, Kristen Lavallee, Heidi J. Lyneham, Patrick Smith, Richard Meiser‐Stedman, Maaike H. Nauta, Ronald M. Rapee, Yasmin Rey, Silvia Schneider, Wendy K. Silverman, Mikael Thastum, Kerstin Thirlwall, Gro Janne Wergeland, Thalia C. Eley, Jennifer L. Hudson

**Affiliations:** ^1^ Department of Psychology Centre for Emotional Health Macquarie University Sydney NSW Australia; ^2^ Department of Biological and Experimental Psychology School of Biological and Chemical Sciences Queen Mary University of London London UK; ^3^ Social, Genetic and Developmental Psychiatry Centre Institute of Psychiatry, Psychology & Neuroscience King's College London London UK; ^4^ NIHR Biomedical Research Centre for Mental Health South London and Maudsley NHS Trust London UK; ^5^ School of Psychology University of Sussex Brighton UK; ^6^ Department of Psychology and Behavioural Sciences Aarhus University Aarhus Denmark; ^7^ Research Institute Child Development and Education University of Amsterdam Amsterdam The Netherlands; ^8^ School of Psychology and Clinical Language Sciences University of Reading Reading UK; ^9^ Department of Psychology Stellenbosch University Stellenbosch South Africa; ^10^ Department of Psychology The University of Cape Town Cape Town South Africa; ^11^ Department of Psychiatry University of Groningen University Medical Center Groningen Groningen The Netherlands; ^12^ Department of Clinical Psychology Faculty of Psychology University of Bergen Bergen Norway; ^13^ Department of Child and Adolescent Psychiatry Anxiety Disorders Research Network Haukeland University Hospital Bergen Norway; ^14^ Department of Psychology University Landau Koblenz Landau Germany; ^15^ Department of Psychology Ruhr‐Universität Bochum Bochum Germany; ^16^ Department of Psychology Institute of Psychiatry King's College London London UK; ^17^ Department of Psychology University of East Anglia Norwich UK; ^18^ Department of Clinical Psychology and Experimental Psychopathology University of Groningen Groningen The Netherlands; ^19^ Department of Psychology Child Anxiety and Phobia Program Florida International University Miami FL USA; ^20^ Child Study Center School of Medicine Yale University New Haven CT USA

**Keywords:** Anxiety, treatment trials, cognitive therapy

## Abstract

**Background:**

Several delivery formats of cognitive behaviour therapy (CBT) for child anxiety have been proposed, however, there is little consensus on the optimal delivery format. The primary goal of this study was to investigate the impact of the child's primary anxiety diagnosis on changes in clinical severity (of the primary problem) during individual CBT, group CBT and guided parent‐led CBT. The secondary goal was to investigate the impact of the child's primary anxiety diagnosis on rates of remission for the three treatment formats.

**Methods:**

A sample of 1,253 children (5–12 years; Mage = 9.3, *SD* = 1.7) was pooled from CBT trials carried out at 10 sites. Children had a primary diagnosis of generalised anxiety disorder (GAD), social anxiety disorder (SoAD), specific phobia (SP) or separation anxiety disorder (SAD). Children and parents completed a semistructured clinical interview to assess the presence and severity of DSM‐IV psychiatric disorders at preintervention, postintervention and follow‐up. Linear mixture modelling was used to evaluate the primary research question and logistic modelling was used to investigate the secondary research question.

**Results:**

In children with primary GAD, SAD or SoAD, there were no significant differences between delivery formats. However, children with primary SP showed significantly larger reductions in clinical severity following individual CBT compared to group CBT and guided parent‐led CBT. The results were mirrored in the analysis of remission responses with the exception that individual CBT was no longer superior to group CBT for children with a primary SP. The difference between individual and group was not significant when follow‐up data were examined separately.

**Conclusions:**

Data show there may be greater clinical benefit by allocating children with a primary SP to individual CBT, although future research on cost‐effectiveness is needed to determine whether the additional clinical benefits justify the additional resources required.

## Introduction

Up to 32% of children and adolescents attending primary care settings present with a primary anxiety disorder (Hansen, Oerbeck, Skirbekk, & Kristensen, 2016; Scott, Mughelli, & Deas, 2005). These problems are highly comorbid with one another (Rapee et al., 2013), and children and adolescents with these problems are at risk of suffering enduring disability. Cognitive behaviour therapy (CBT) is regarded as the front‐line psychological treatment for child anxiety with approximately 60% of children in remission from their anxiety disorder diagnosis immediately after completing treatment (James, James, Cowdrey, Soler, & Choke, 2015). However, despite widespread support for CBT, the empirical data clearly indicate that a sizeable proportion of children do not recover. A clearer understanding of the limits to CBT, and correspondingly, the ideal conditions for delivery of CBT for child anxiety are essential. There are now a variety of evidence‐based CBT programs defined under the broad term ‘CBT’. Some of the main programs in the field are trans‐diagnostic, whereas other are disorder‐specific (Schneider et al., 2013), or intensive treatments (Ollendick et al., 2009). Even though programs share some important similarities, they also differ in a number of areas; for example, duration, treatment targets and level of parental involvement.

### Efficacy of CBT treatment formats

Decisions regarding the best way to allocate children to child anxiety treatments are currently poorly understood. Our goal in the current study was to investigate whether clinical responses to individual CBT, group CBT and guided parent‐led CBT are influenced by the child's primary anxiety diagnosis.^1^ There are very few published evaluations of the costs of CBT treatments for child anxiety, but it is typically presumed that whilst individual CBT allows for extensive tailoring of treatments to the individual, it is most expensive. Group CBT may be slightly cheaper and there are invaluable opportunities for peer normalisation, positive peer modelling, reinforcement and social support (Manassis et al., 2002). Three trials have concluded that treatment responses were no different for individual CBT and group CBT (Liber et al., 2008; Manassis et al., 2002; Wergeland et al., 2014). In contrast, one meta‐analysis showed that individual CBT for child anxiety led to superior effect sizes (on a child reported self‐report symptom measure) compared to group CBT (Reynolds, Wilson, Austin, & Hooper, 2012). Guided parent‐led CBT (i.e. delivered directly to children via their parents) can be delivered remotely thereby increasing the chances that it is cost‐effective (Creswell et al., 2017; Lyneham & Rapee, 2006; Silverman, Pettit, & Lebowitz, 2016; Thirlwall et al., 2013). A recent Cochrane review indicated that there were no differences in the efficacy of individual CBT, group CBT and family/parental CBT^2^ (James et al., 2015).

Despite having important implications for clinical decision making, previous meta‐analyses (James et al., 2015; Reynolds et al., 2012) are yet to elucidate if there is a clinical need to allocate children presenting with a primary anxiety problem to a particular treatment format. There are very plausible reasons that there could be disorder‐specific responses to different treatment formats. For example, in social anxiety disorder (SoAD) the group situation may provide important opportunities for practicing exposure skills, but it could also prove to be too anxiety provoking, making it difficult for children to engage with their therapist and learn core CBT skills. It may be more difficult to deliver group CBT to children with a specific phobia (SP) due to the amount of psychoeducation and in‐session exposure typically needed to treat these cases. As such, establishing whether individual CBT, group CBT or guided parent‐led CBT leads to similar improvements for the range of child anxiety disorders is an empirical question with important implications for the development of stepped care models.

### Diagnosis and clinical responses to CBT treatment formats

Three small RCTs (Liber et al., 2008; Manassis et al., 2002; Wergeland et al., 2014) have investigated the impact of having an SoAD diagnosis on clinical outcomes following group or individual therapy. Manassis et al. (2002) reported a significant interaction effect between type of anxiety disorder and treatment approach in that children with high levels of social anxiety symptoms improved significantly more with individual CBT compared with group CBT. A second trial showed no difference in outcomes for children with a primary SoAD (Wergeland et al., 2014). A third trial showed that when the child had an SoAD diagnosis in their profile, greater reductions in internalising symptoms (according to father's report) were achieved with group CBT compared to those achieved by individual CBT (Liber et al., 2008). These mixed findings are at odds with some findings in the adult SoAD field that have shown individual CBT is associated with more improvement in clinical severity than group CBT (Ingul, Aune, & Nordahl, 2014; Stangier, Heidenreich, Peitz, Lauterbach, & Clark, 2003). Taken together, these findings underscore the need to investigate interactions between treatment format and diagnosis in a large well‐powered sample.

The literature on the differential effect of individual CBT and group CBT for response to different CBT treatment formats in generalised anxiety disorder (GAD), SP and separation anxiety disorder (SAD) is sparse. Manassis et al. (2002) found greater reductions in mother‐rated anxiety symptoms for children with a primary GAD across both formats compared with children with SAD, SoAD or SP. Another study found that participants with a primary GAD diagnosis showed significantly greater reductions in parent‐rated anxiety and depressive symptoms in individual CBT relative to group CBT, but children with SAD benefitted equally (Wergeland et al. (2014). The only trial that evaluated the impact of having a primary SP on treatment responses found no differences between individual CBT and group CBT (Manassis et al., 2002). Reasons contributing to the variable pattern of results include the fact that treatment responses were not always evaluated with respect to changes in symptom severity and remission (Liber et al., 2008; Manassis et al., 2002). Some studies assessed differences across primary problems (Wergeland et al., 2014), whereas others looked at differences according to having a diagnosis in the overall profile (Liber et al., 2008), some trials had very small sample sizes that limited conclusions (Liber et al., 2008; Manassis et al., 2002), and all previous studies have used either a parent‐ or child‐rated self‐report measure to measure symptom change.

The present study investigated whether clinical responses to treatment formats are influenced by the child's primary anxiety diagnosis. This will be the first comparison of individual CBT, group CBT and guided parent‐led CBT within individual anxiety disorder categories in the child anxiety field. We used the Genes for Treatment dataset, which is a pooled sample of children taking part in CBT treatments for child anxiety (Hudson et al., 2015; Keers et al., 2016). The dataset employed in this study was significantly larger than previous RCT's that have assessed these questions in the field. The use of pooled individual‐level data in this study allows for greater precision when controlling for covariates and in the examination of potential treatment moderators. This is also the first study to evaluate treatment responses in a robust manner using a clinician‐rated composite measure derived from ratings on a structured clinical interview. Children were split into diagnostic groups based on their primary anxiety diagnosis. This is very relevant to both research and routine clinical practice where it is commonplace for the clinician to assess all presenting problems and focus treatment on the disorder leading to the highest levels of impairment.

The primary aim of this study was to investigate the changes in clinical severity associated with individual CBT, group CBT and parent‐led CBT in four separate primary anxiety disorder categories ‐ SoAD, GAD, SAD and SP. The secondary aim was to assess these questions using rates of remission (i.e. absence/presence of the primary diagnosis at the end of treatment) as the core dependent variable of interest. If significant relationships were found in the main analyses, interactions between diagnosis and treatment format would be explored. The current state of the literature precluded any a priori hypotheses regarding these interactions in the present study.

## Method

### Sample

Data for 1,253 children^3^ aged between 5 and 12 years (M_age_ = 9.3, *SD* = 1.7) was pooled from centres in Sydney, Australia (*n* = 619; 49.40%) Reading, United Kingdom (*n* = 299; 23.86%), Bergen, Norway (*n* = 84; 6.70%), Aarhus, Denmark (*n* = 82; 6.54%), Bochum, Germany (*n* = 38; 3.03%), Basel, Switzerland (*n* = 48; 3.83%), Miami, Florida (*n* = 40; 3.19%), Groningen, the Netherlands (*n* = 21; 1.68%), Oxford (*n* = 20; 1.60%) and Amsterdam, the Netherlands (*n* = 2; 0.16%). The process of patient recruitment and the pooling of data has been described in greater detail elsewhere (Hudson et al., 2015; Lester et al., 2016; Roberts et al., 2015).

The inclusion criteria were: meeting DSM‐IV criteria for a primary diagnosis of GAD, SoAD, SP or SAD based on the composite report of the parent and the child; having a sample of DNA available for analysis, and taking part in a manualised CBT treatment protocol. Exclusion criteria were a diagnosis of significant intellectual impairment, a neurological disorder or psychosis for the child.

Each study received ethical approval from their local hospital/university recruitment site. Informed consent was obtained from adult carers/parents and assent from young people. The demographic characteristics of the sample are presented in Table 1. A description of the interventions carried out at the different sites is reported elsewhere (Hudson et al., 2015) and is summarized in Appendix S1.

**Table 1:** Demographic and sample characteristics across diagnosis

	GAD	SoAD	SP	SAD	Total	Test statistic	Differences
% of total sample (*N*)	40.5 (507)	22.6 (283)	11.4 (143)	25.5 (319)	100 (1,253)	*χ* ^2^ (3) = 216.63, *p *<* *.001	–
% Female participants (*n*)	48.9 (248)	45.2 (128)	53.8 (77)	55.2 (176)	50.2 (629)	*H* (3) = 7.04, *p *=* *.07	–
Age (*M*,* SD*)	9.4 (1.7)	9.5 (1.6)	9.4 (1.6)	9.0 (1.7)	9.3 (1.7)	*F* (3) = 4.9, *p *=* *.002	GAD, SoAD, SP > SAD
CSR severity at baseline (*M*,* SD*)	6.2 (.94)	6.1 (1.0)	6.4 (1.1)	6.3 (1.0)	6.1 (1.0)	*F* (3) = 2.4, *p *=* *.17	–
% Comorbid externalising disorder (*n*)	21.8 (107)	21.1 (53)	8.4 (19)	20.8 (47)	19.4 (226)	*H* (3) = 7.04, *p *=* *.07	–
% Comorbid internalising disorder^a^ (*n*)	10.6 (52)	13.6 (34)	3.5 (5)	6.7 (19)	9.4 (110)	*H* (3) = 14.1, *p = *.003	SoAD > SP, SAD
% CBT treatment (*n*)							
Individual (*n*)	15.3 (78)	29.3 (83)	32.2 (46)	42.0 (134)	27.2 (341)	*H* (3) = 73.6, *p < *.001	SoAD, SP, SAD > GAD SAD > SoAD, SP
Group (*n*)	65.7 (333)	50.2 (142)	41.3 (59)	37.0 (118)	52.1 (652)	*H* (3) = 73.1, *p < *.001	GAD > SoAD, SP, SAD SoAD > SAD
Parent‐led (*n*)	19.1 (97)	20.5 (58)	26.6 (38)	21.0 (67)	20.8 (260)	*H* (3) = 14.1, *p = *.28	–

GAD, generalised anxiety disorder; SoAD, social anxiety disorder; SP, specific phobia; SAD, separation anxiety disorder; CSR, Clinician severity rating score.

a Comorbid internalising problem refers to a nonanxious internalising disorder.

### Measures

The DSM‐IV anxiety disorders were measured pre‐ and postintervention via semistructured clinical interviews carried out with parents and children. Across individual trials, follow‐up data were collected at variable time points (3, 6 or 12 months). All interviews were carried out by graduate level staff or trainee psychologists.

The primary outcome, absolute change in clinical severity of the child's primary problem across treatment, was established using clinician severity rating (CSR) scores on the Anxiety Disorders Interview Schedule Child/Parent (ADIS‐C/P; Silverman & Albano, 1996). In giving a CSR rating, the assessor makes a single assessment of function on a scale from 0 (absent) to 8 (very severely disturbing/disabling) by considering the symptom severity, avoidance and interference with the child's functioning in core areas (e.g. school, family, friends, peers). The secondary outcome, rates of remission (absence of primary diagnosis) following treatment was determined according to whether the child endorsed the requisite symptoms in line with the DSM criteria measured on the ADIS‐C/P (Silverman & Albano, 1996). A child's diagnosis was assumed to be in remission if he/she no longer presented with the requisite symptoms required for the DSM diagnosis, which corresponds to a CSR score of <4.

The vast majority of postassessments were blinded, although for some trials and a minority of nontrial cases in the sample this was not possible (see supplementary materials in Appendix S1 for references to individual trials and a description of methods). Inter‐rater reliability was assessed and found to be satisfactory (see supplementary details in Appendix S1 for references to individual trials).

In Bochum, the diagnosis was made using the *Diagnostisches Interview bei psychischen Strungen im Kindes‐ und Jugendalter* (Kinder‐DIPS; Neuschwander, In‐Albon, Adornetto, Roth, & Schneider, 2013; Schneider, Unnewehr, & Margraf, 2009). The Kinder‐DIPS is based on the ADIS‐C/P and provides the same data as the ADIS.

### Data analysis

All analyses were conducted in STATA Version 14 (StatCorp, College Station, TX). As we were most interested in differences in the efficacy of treatment formats within each disorder, linear mixture models were carried out separately for each diagnostic category. The primary dependent variable of interest was the absolute change in clinical severity of the primary anxiety problem. The secondary aim was to assess these questions using rates of remission (i.e. absence/presence of the primary diagnosis at the end of treatment) as the core‐dependent variable of interest. The main analysis was carried out using outcome data pooled across post and follow‐up time points (3, 6 or 12 months) as the dataset was underpowered to examine fixed effects across time points due to the variable follow‐up time points (i.e. 3, 6 and 12 months) employed in individual studies.

Absolute changes in clinical severity and remission rates during the study period, and the extent of changes between diagnostic categories for the Genes for Treatment sample, have previously been reported (Hudson et al., 2015). A preliminary analysis was carried out to show the results of this previous study are replicated in this subsample of Genes for Treatment dataset. A Linear Mixture Model was fitted using the linear and quadratic effects of time, age, gender, baseline severity, treatment format and diagnostic category as fixed effects and clinical severity (i.e. CSR scores) as the dependant variable. In a second similar set of analyses, a logistic regression model was fitted with the same fixed effects and the dependent variable was the presence/absence of the primary anxiety disorder diagnosis.

For the main analyses investigating diagnosis‐specific effects, four separate Linear Mixture Models were carried out to investigate the primary research questions regarding the efficacy of treatment format within each diagnostic category (GAD, SoAD, SP and SAD). All models included individual random intercepts in order to account for the correlation of repeated measures. Trials were nested within sites. Trial was entered in the model as a higher order random effect to account for possible differences by trial or site. The model included the linear and quadratic effects of time, age, gender, baseline severity, treatment format and diagnostic category as fixed effects and CSR scores as the dependant variable of interest. Supplementary analyses are also presented showing outcomes for these analyses at post and follow‐up time points. In a second similar set of analyses, we used logistic regression models to investigate remission of the primary diagnosis following treatment in each of the four diagnostic categories.

Bonferroni corrections were employed and a *p* value of at least .006 (.05/8) was set on each of the linear models to indicate a significant result, based on eight analyses (four linear regression models, four logistic regression models) being conducted in the main analyses.

In addition to the separate analyses above for each diagnosis, we also conducted a formal test of the interaction between diagnosis and treatment format on clinical severity with the whole sample. Interactions were only investigated within a diagnostic category if a significant result was found in the main results. Given the exploratory nature of these analyses, a *p* value of <.05 was set to indicate significance. To determine whether there was an interaction of disorder and treatment format on change in clinical severity and remission rates, variables were dummy coded (SP vs. Other anxiety disorders, individual CBT vs. Other CBT treatments) and then entered as covariates in the model. Due to the nonrandomised nature of the study, we also carried out a post‐hoc logistic regression analysis to see whether the proportions of children allocated to treatment formats significantly differed within each diagnostic category.

## Results

### Sample characteristics

Table 1 shows the baseline descriptive characteristics across the four diagnostic categories. The majority of the sample had a primary diagnosis of GAD (*n* = 508), followed by SAD (*n* = 319), SoAD (*n* = 283) and finally SP (*n* = 143). Across the samples, 9.4% of children had a comorbid internalising disorder (a nonanxiety internalising disorder) and 19.4% of children had a comorbid externalising disorder. The most common treatment format was group CBT (52.0%; *n* = 652), followed by individual CBT (27.2%; *n* = 341) and then guided parent‐led CBT (20. 8%; *n* = 260). The proportion of cases at each site within each treatment format for each primary diagnostic category is reported in Table S1.

Average CSR severity scores ranged from 6.1 (*SD* = 1.0) to 6.4 (*SD* = 1.1) across categories. The proportions of females in the four groups ranged from 45.2% in primary SoAD to 55.2% in children with primary SAD. The mean age of children across diagnostic categories ranged from 9.0 years in the SAD group to 9.5 years in the SoAD group. Of note, children with primary SPs were the least likely to have a comorbid nonanxiety internalising problem (3.5%) and externalising problem (8.4%). In contrast, rates of comorbid externalising problems were highest in the GAD group (21.8%) and rates of comorbid nonanxiety internalising problems were highest in the SoAD group (13.6%).

### Changes in clinical severity and remission rates for CBT treatment in the full sample

As reported previously (Hudson et al., 2015), there was a significant reduction in clinical severity scores (i.e. CSR scores; *p *<* *.001) during the study period (Table S2). Children with a primary SoAD (*p *<* *.001) diagnosis had significantly smaller reductions in clinical severity during the study period relative to GAD (*p *<* *.001), SP (*p* = .001), and SAD (*p *<* *.001). Reductions in clinical severity were comparable for GAD, SP and SAD (Table S2). Similarly, for the full sample there were improvements in remission rates (*p *<* *.001) during the study period (Table S2). Children with a primary SoAD (*p *<* *.001) diagnosis were also less likely to lose their diagnosis relative to GAD (*p *<* *.001), SP (*p = *.003) and SAD (*p *<* *.001). Improvements in remission rates were comparable for GAD, SP and SAD.

### Change in symptom level and remission rates for CBT treatment formats across primary anxiety disorder categories

Table 2 summarises the results of linear mixed models evaluating changes in CSR scores over treatment formats for the four diagnostic categories. Treatment format did not have a significant effect on changes in clinical severity for those with a primary diagnosis of GAD, SoAD or SAD. These findings were replicated in supplementary analyses carried out on response data when postintervention (Tables S4a and S4b) and follow‐up (Tables S5a and S5b) data were considered separately.

**Table 2:** Results of linear mixed models examining the relationship between primary diagnosis and treatment format on changes in clinical severity (CSR) using all follow‐up points

	GAD		SoAD		SP		SAD	
	β (*SE*)	95% CI	β (*SE*)	95% CI	β (*SE*)	95% CI	β (*SE*)	95% CI
Severity of primary diagnosis at baseline^a^	.12^d^ (0.03)	[0.06, 0.19]	.25^d^ (0.04)	[0.17, 0.33]	.06 (0.06)	[−0.06, 0.18]	.21^d^ (0.05)	[0.12, 0.30]
CBT treatment								
Individual	b	b	b	b	b	b	b	b
Group‐ based^c^	.10 (0.16)	[−0.20, 0.41]	.09 (0.16)	[−0.22, 0.41]	.63^d^ (0.15)	[0.33, 0.93]	.18 (0.16)	[−0.15, 0.50]
Guided parent‐led^c^	−.19 (0.18)	[−0.55, 0.17]	−.13 (0.20)	[−0.52, 0.27]	.61^d^ (0.17)	[0.27, 0.95]	.23 (0.21)	[−0.17, 0.64]
Age	−.01 (0.02)	[−0.04, 0.02]	−.00 (0.03)	[−0.05, 0.05]	.05 (0.03)	[−0.03, 0.13]	.00 (0.03)	[−0.05, 0.05]
Gender	.13 (0.08)	[−0.02, 0.28]	.13 (0.08)	[−0.02, 0.28]	−.02 (0.12)	[−0.26, 0.22]	.12 (0.09)	[−0.05, 0.29]

GAD, generalised anxiety disorder; SoAD, social anxiety disorder; SP, specific phobia; SAD, separation anxiety disorder; CBT, cognitive behaviour therapy.

a Standardised regression coefficients (β) significantly different than zero indicate association with clinical severity after treatment.

b Reference category.

c Standardised regression coefficients (β) significantly different than zero indicate higher (negative value) or lower (positive value) changes in clinical severity compared to the reference category.

d *p < .006.

**Figure 1:**
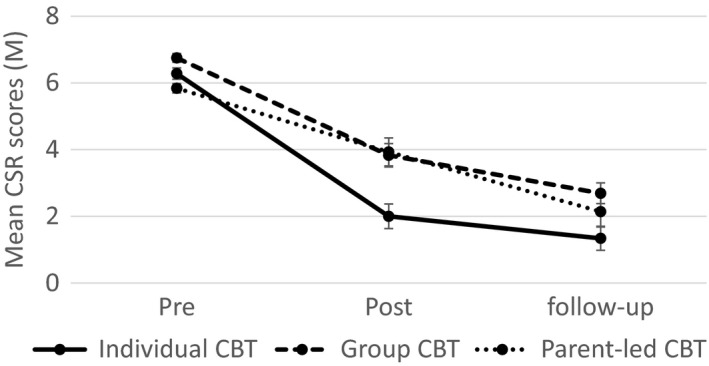
Mean clinician severity rating (CSR) scores over time across individual cognitive behaviour therapy (CBT), group CBT and parent‐led CBT for children with a primary specific phobia. Post and follow‐up means include covariates used in main analysis

However, treatment format *was* significantly associated with outcomes for children with SP. Individual CBT was associated with significantly larger improvements in clinical severity than both group CBT (*p < *.001) and guided parent‐led CBT (*p < *.001). Figure 1 shows the change in CSR scores for children with a primary SP over the three assessment time points in each of these treatment formats. These results suggest a 1.58‐point greater reduction in CSR scores for individual CBT over group CBT and a 1.54‐point greater reduction in CSR scores for individual CBT in comparison to guided parent‐led CBT.

This result remained when outcomes were limited to the post‐treatment time point (Table S4a). At follow‐up, individual CBT was no longer more effective than guided parent‐led CBT (*p *=* *.17) or group CBT (*p *=* *.007) once Bonferroni corrections were employed. This provides some evidence that the significant advantage of individual CBT over both treatments reduces at follow‐up, although this finding should be interpreted cautiously in light of the smaller sample size at the follow‐up analysis.

A similar pattern of results was observed in the analysis of remission. Table 3 summarises the results of logistic mixed models examining remission rates over the three treatment formats within each psychiatric condition. The mean severity sores of the three treatment formats within each psychiatrist condition are presented in Table S3. Treatment format did not have a significant effect on improvements in remission for GAD, SoAD and SAD. In contrast, children with a primary SP who received individual CBT were significantly more likely to lose their SP diagnosis compared to children in guided parent‐led CBT (*p *=* *.005). There were no significant differences in remission rates for individual CBT and group CBT (*p = *.04) after correction for multiple testing. This finding was replicated in the supplementary analysis carried out on the postintervention data (Table S4b). There were no significant differences in remission rates between individual CBT, group CBT and guided parent‐led CBT for SP when the follow‐up data were analysed separately (Table S5b).

**Table 3:** Results of logistic mixed models examining the relationship between primary diagnosis and treatment format on change in diagnostic frequency (remission) using all follow‐up points

	GAD		SoAD		SP		SAD	
	OR (*SE*)	95% CI	OR (*SE*)	95% CI	OR (*SE*)	95% CI	OR (*SE*)	95% CI
Severity of primary diagnosis at baseline^a^	0.75 (0.13)	[0.52, 1.0]	0.45^d^ (0.09)	[0.30, 0.68]	0.70 (0.22)	[0.38, 1.3]	0.35^d^ (0.19)	[0.21, 0.61]
CBT treatment								
Individual	b	b	b	b	b	b	b	b
Group‐based^c^	0.75 (0.61)	[0.15, 30.7]	0.64 (0.46)	[0.16, 20.6]	0.15 (0.15)	[0.02, 10.0]	0.47 (0.42)	[0.08, 20.7]
Guided parent‐led^c^	10.4 (10.4)	[0.20, 90.5]	0.70 (0.65)	[0.11, 40.3]	0.05^d^ (0.05)	[0.01, 0.32]	0.08 (0.09)	[0.01, 0.69]
Age	10.0 (0.09)	[0.88, 10.2]	0.94 (0.11)	[0.74, 10.2]	0.87 (0.16)	[0.60, 10.3]	10.0 (0.15)	[0.77, 10.4]
Gender	0.66 (0.19)	[0.37, 10.2]	0.82 (0.29)	[0.41, 10.6]	10.7 (0.97)	[0.54, 50.2]	0.43 (0.20)	[0.17, 10.1]

GAD, generalised anxiety disorder; SoAD, social anxiety disorder; SP, specific phobia; SAD, separation anxiety disorder; CBT, cognitive behaviour therapy.

a Odds ratios of variables predicting a higher likelihood of remission are significantly greater than one, whereas variables predicting a lower likelihood of remission have odds ratios of significantly <1.

b Reference category.

c Odds ratios of variables predicting a higher likelihood of remission relative to the reference category are greater than one, whereas variables predicting a lower likelihood of remission relative to the reference category have odds ratios of <1.

d *p* < .006.

**Figure 2:**
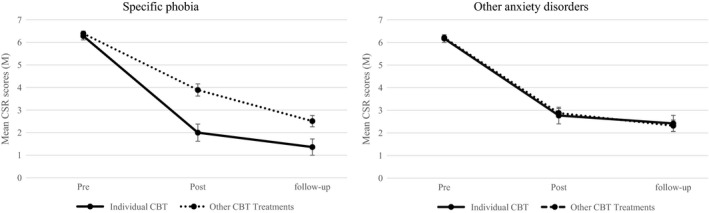
Mean clinician severity rating (CSR) scores over time for individual cognitive behaviour therapy relative to other treatments for children with a primary specific phobia relative to children with other primary disorders. Post and follow‐up means include covariates from main analysis

To determine whether there was a diagnosis (SP vs. Other Anxiety Disorders) by treatment format (Individual CBT vs. Other CBT treatments) interaction on clinical severity, variables were dummy coded and then entered as covariates in the regression model. Preliminary analysis of baseline differences between SP and Other Anxiety Disorders groups showed there were no differences with respect to baseline CSR scores, age, gender or presence of a comorbid externalising problems (*p* > .05). Children in the SP group were less likely than children in the other categories to have a comorbid nonanxious internalising problem (*χ* ^2^ (1) = 6.59 *p *=* *.01), however, this variable was not controlled for in the analysis as a high correlation of anxiety and mood problems in youth is expected, and partialling out this variable may remove an important element of the anxiety construct of interest. The statistical interaction between diagnosis (SP vs. Other Anxiety Disorders) and treatment format (Individual CBT vs. Other CBT treatments) on CSR scores was significant (*p = *.003; based on a threshold of *p *<* *.05). Figure 2 summarises this interaction. Individual CBT was more effective than the other treatments for children with primary SP (β* *= .63; CI_95_: [0.35, 0.89]; *p *<* *.001). In contrast, individual CBT was no more effective than other treatment formats for children with other primary anxiety disorders (β* *= −.04; CI_95_: [−0.24, 0.16]; *p *=* *.71). Post‐hoc analyses also suggested this result was not an artefact of biases in treatment allocation. Children with a primary SP were no more likely to receive individual CBT compared to any other sort of treatment (β* *= .33; CI_95_: [−0.02, 0.68]; *p *=* *.07). The same pattern of significant and nonsignificant comparisons was replicated when remission rates were investigated.

## Discussion

In the present study, we evaluated the treatment responses associated with individual CBT, group CBT and guided parent‐led CBT in a pooled sample of children with a primary anxiety disorder diagnosis. Our results indicated there were no differences across the three treatment formats for children with primary SoAD, GAD or SAD. However, individual CBT was better than guided parent‐led CBT for SP in terms of both improvement in symptoms and remission rates. Individual CBT also performed better than group CBT for SP in the clinical severity change analysis, but not in the remission analysis (after correction for multiple testing). The interaction between diagnosis (SP vs. Other anxiety disorders) and treatment format (Individual CBT vs. Other CBT treatments) on changes in clinical severity was significant, with the analyses suggesting this was not due to baseline severity of symptoms, site differences, or biases in treatment allocation.

The inability to replicate the significant differences between individual and group CBT for SP in the remission model could be explained by the fact that remission outcomes rely on cutoffs and are less well powered. Our approach of applying Bonferroni corrections to control for multiple comparisons may have been overly restrictive. This finding is also at odds with the Manassis et al. (2002) study, which was limited by its small sample size. Whilst the result found here was obtained in a large sample that used a clinician‐rated measure, it is also important to acknowledge that our pooled analysis did not have the same tight experimental controls as a single RCT. In the present study, the statistics performed on the primary outcome of interest are based on pooled data from postintervention and follow‐up measures time points (3, 6 and 12 months), meaning that the time point at which the significant effects took place in this study is less clear than in the Manassis et al. (2002) study. The difference between individual and group for SP was observed when examining postdata separately but not follow‐up data. These differences also offer potential explanations for the discrepant results across the two studies.

Our results suggest that children with SPs can improve in parent‐led, group and individual CBT, but they do significantly better in individual CBT. It was particularly noteworthy that although SP tends to be considered as less pervasive and more amenable to treatment, individual CBT produced better outcomes than either group CBT or guided parent‐led approaches. Children present with a range of SP's in clinical settings. In individual CBT, therapists tailor protocols by providing specific psychoeducation surrounding the fear (e.g. education about dog safety) as well as in‐session‐guided exposure to feared situations, and training parents to be efficacious in guiding their child through the exposure process. In group CBT and guided parent‐led CBT, such specificity to target avoidance behaviours may not be possible, and this explanation may account for the superiority of individual CBT here. The data here show a stronger clinical benefit is associated with the allocation of children with SPs to individual CBT. However, decisions regarding the allocation of children to a treatment format are complex, and influenced by multiple factors including pragmatic concerns (e.g. ability of parents to bring child to appointments, demand for service, waiting lists etc.), patient preferences and therapist preferences. Furthermore, an analysis of the cost‐effectiveness of individual CBT for SP relative to the other treatment formats is needed, given the additional resources required per patient to deliver individual CBT.

These results are consistent with previous studies showing individual CBT and group CBT are comparable for GAD (Manassis et al., 2002), SoAD (Wergeland et al., 2014) and SAD (Wergeland et al., 2014). Our findings are at odds with other studies that found outcomes with individual CBT to be better than those with group CBT for children with GAD (Wergeland et al., 2014) and with SoAD (Liber et al., 2008; Manassis et al., 2002). There could be numerous reasons for this, including differences in sample size, study design (outlined previously) and differences in the measures used to index dependant variables of interest. The finding that children with primary SoAD, GAD and SAD respond similarly, regardless of treatment format implies that cheaper treatments can be considered as a first line approach for these disorders.

Some aspects of the present methodology merit comment. Firstly, this sample was part of the Genes for Treatment study and children could only be included in the sample if they gave a saliva sample. As such, there may be distinct differences in the characteristics of children whose parents have consented to provide a sample of their child's saliva for research purposes. Second, the majority of programs included in this analysis were also trans‐diagnostic in nature. It is unclear whether the same pattern of findings would be obtained if disorder‐specific protocols were tested in samples of children with heterogeneous psychiatric presentations. Third, online self‐help programs are now a very common low intensity self‐help approach, but these participants were excluded due to their low frequency in the sample. A future study comparing ‘Internet delivery to the three treatment formats examined in this study is needed to give a definitive answer regarding the utility of low intensity approaches. There are also some statistical considerations worth noting. Group sizes were uneven, with the fewest number of cases found in the SP group (*N* = 158 cases) and a larger group may have produced a different pattern of results. While SP participants came from eight sites, not all sites performed all protocols, so there might still be a lingering effect of site, despite controlling for this statistically. The results of the logistic regressions carried out on follow‐up data must be interpreted cautiously as the wide confidence intervals on a number of results indicated the analysis was underpowered. Finally, the analysis of follow‐up data separately from post‐treatment data was underpowered in this study.

There are some important avenues for future research. An important next step will be to compare the efficacy of individual CBT with the widely used one‐session CBT treatments for phobic youth (Ollendick et al., 2009). As our pooling approach affords less experimental control than can be gleaned from a single RCT, there is a need for large‐scale randomised controlled trials with long‐term follow‐up periods addressing differences in treatment format. These studies should also measure important cognitive and biological processes that could be driving differential responses to CBT treatment formats. Finally, a study to assess these questions among adolescents is needed.

This is the first study to compare outcomes from guided parent‐led CBT, group CBT and individual CBT among children with four primary anxiety disorder diagnoses. Individual CBT was superior to group CBT and guided parent‐led CBT for children with a primary SP. With variable CBT programs being delivered in the community, further research is needed to understand the optimal conditions for allocating anxious children to different forms of CBT.

**Key points**

- The impact of CBT treatment format on response to treatment was investigated in a sample of children with a primary GAD, SAD, SP and SoAD.
- In children with a primary GAD, SAD or SoAD, there were no differences between individual CBT, group CBT and guided parent‐led CBT.
- In children with a primary SP, individual CBT led to greater reductions in clinical severity and higher remission rates than guided parent‐led CBT.
- In children with a primary SP, individual CBT led to greater reductions in the clinical severity of primary problems than group CBT.
- Allocation of children with SPs to individual CBT may have a stronger clinical benefit than allocation to group or guided parent‐led CBT.
- For children with GAD, SoAD and SAD decisions about treatment format can incorporate cost considerations and patient choice.

## Supporting information


**Appendix S1.** References to individual trials and a description of methods.
Table S1. Percentage of cases per site for treatment formats within diagnostic categories.
**Table S2.** Results of linear and logistic mixed models examining the relationship between primary diagnosis and treatment format in the full sample using all follow‐up points.
**Table S3.** Means (standard errors) of the relationship between primary diagnosis and treatment format on severity scores (CSR) at pre, post and follow‐up.
**Table S4a.** Results of linear mixed models examining the relationship between primary diagnosis and treatment format on severity scores (CSR) using the post time point only.
**Table S4b.** Results of logistic mixed models examining the relationship between primary diagnosis and treatment format on severity scores (CSR) using the post time point only.
**Table S5a.** Results of linear mixed models examining the relationship between primary diagnosis and treatment format on severity scores (CSR) using the follow‐up time point only.
**Table S5b.** Results of logistic mixed models examining the relationship between primary diagnosis and treatment format on severity scores (CSR) using the follow‐up time point only.
Figure S1. Mean clinician severity rating (CSR) scores over time across individual CBT, group CBT and parent‐led CBT for children with GAD, SAD, SoAD.

## Notes

1 The primary problem is the disorder deemed by the clinician to be the most impairing of all the child's psychiatric diagnoses. This judgement is made by consideration of symptom severity, avoidance, and interference with core aspects of functioning for each psychiatric diagnosis.

2 In this study, ‘family/parental CBT’ was defined as treatment with the direct involvement of parents. In some instances, the whole family was involved, in others the parents were present for conjoint or separate sessions, and sometimes the parents were cotherapists.

3 Please note, whilst there were some cases taking part in computer/internet CBT and some children with additional primary disorders (e.g. obsessive compulsive disorder, posttraumatic stress disorder, etc.) within the Genes for Treatment dataset, these occurred with low frequency in the dataset and were hence excluded from the analysis. Additionally, all adolescents were excluded from the dataset as there were no adolescents that took part in parent‐led CBT.
