# Supplementary material for: The impact of treatment delivery format on response to cognitive behaviour therapy for preadolescent children with anxiety disorders
Source: J Child Psychol Psychiatry. 2018 Mar 9;59(7):763–72. doi: 10.1111/jcpp.12872 (PMC6055633; doi:10.1111/jcpp.12872)
Supplement: Supplementary file 1 — Appendix S1. References to individual trials and a description of methods. Table S1. Percentage of cases per site for treatment formats within diagnostic categories. Table S2. Results of linear and logistic mixed models examining the relationship between primary diagnosis and treatment format in the full sample using all follow‐up points. Table S3. Means (standard errors) of the relationship between primary diagnosis and treatment format on severity scores (CSR) at pre, post and follow‐up. Table S4a. Results of linear mixed models examining the relationship between primary diagnosis and treatment format on severity scores (CSR) using the post time point only. Table S4b. Results of logistic mixed models examining the relationship between primary diagnosis and treatment format on severity scores (CSR) using the post time point only. Table S5a. Results of linear mixed models examining the relationship between primary diagnosis and treatment format on severity scores (CSR) using the follow‐up time point only. Table S5b. Results of logistic mixed models examining the relationship between primary diagnosis and treatment format on severity scores (CSR) using the follow‐up time point only. Figure S1. Mean clinician severity rating (CSR) scores over time across individual CBT, group CBT and parent‐led CBT for children with GAD, SAD, SoAD. [file JCPP-59-763-s001.docx]

**Additional Supporting information for*:* The impact of treatment delivery format on response to cognitive behaviour therapy for pre-adolescent children with anxiety disorders – by Jennifer L. Hudson & Thalia C. Eley et al.**

**Appendix S1.** References to individual trials and a description of methods.

**Method**

Unless otherwise specified, clinical trials included all primary anxiety disorder diagnoses. All sites made secondary anxiety disorder diagnoses where appropriate. According to the categories determined by [Manassis et al. (2014)](#_ENREF_13), the first author coded the type of parental involvement in the treatment offered at each site, confirmed by the clinical leads from each site. The type of parental involvement was coded into the following three categories: 1) low parental involvement: parents involved in less than 50% of sessions or for a brief time at the beginning of each session; 2) active parental involvement + low contingency management (CM)/transfer of control (TC): parents attended more than 50% of sessions, but parent involvement did not involve a focus on CM or TC. For example, parental involvement focused on parent/child interactions or parent anxiety or parenting but did not use contingency management or a model by which the therapist gradually transferred control to the parent; 3) active parental involvement  + high CM/TC: parents attended more than 50% of sessions and included a focus on CM or TC (e.g., parental involvement focused on teaching parents to reward children for facing anxiety-provoking situations and/or the therapy used a model by which the  therapist gradually transferred control to the parent.

**Sydney, Australia** (n **=** 706). Participants aged 6–18 were recruited from the Centre for Emotional Health, Macquarie University, Sydney. All participants completed the Cool Kids Program ([Rapee et al., 2006a](#_ENREF_16)), with 9–12 family sessions involving the parents (the majority of which were conducted in groups; 8% of the sample’s DNA were collected retrospectively). Variations on this treatment program include a subgroup from previous randomized trials who received group, individual, or phone-based cognitive-behavioural therapy (CBT) sessions ([Hudson et al., 2009](#_ENREF_9); [Rapee, Abbott, & Lyneham, 2006b](#_ENREF_17)); participants from a guided self-help trial with phone support for children in rural Australia ([Lyneham & Rapee, 2006](#_ENREF_12)); a group from a trial with additional parental anxiety management ([Hudson et al., 2013](#_ENREF_8)); and those recruited from an ongoing randomized trial of progressive allocation to treatment (stepped care). Therapists from these trials included a mix of clinical psychology master’s students and experienced therapists. In the stepped care trial, guided self-help was led by four-year trained psychologists, and the final step was delivered by experienced clinicians. Otherwise, therapy was delivered by clinical psychology graduate students. Treatments were coded as active parental involvement with high CM/TC. None of the trials were registered. Children on medication for anxiety and depression were included if their medications were kept stable during the trial. Children taking ADHD medications were also permitted entry into the trial. Post assessments for all cases recruited to take part in trials were carried out by blinded, independent assessors.

**Reading** (n = 340) **and Oxford** (n = 21). Participants aged 5–18 were recruited jointly from Reading and Oxford from eight trials at the Berkshire Child Anxiety Clinic (University of Reading) and the Oxfordshire Primary Child and Adolescent Mental Health Service. Participants received treatment in three main themes; one focusing on children with anxious mothers; a set of trials using a parent-guided self-help CBT program; and an online CBT program for adolescents.

*The Mother and Child (MaCh) project.* ([Creswell et al., 2007](#_ENREF_3)) Children whose mother also had a current anxiety disorder completed an 8-session manual-based CBT treatment based on the Cool Kids Program ([Lyneham, Abbott, Wignall, & Rapee, 2003](#_ENREF_11)). The mothers of these children also received extra sessions focusing on their own anxiety and on mother–child interactions. Therapists in this trial had several years of experience. The trial was registered with the ISRCTN registry (19762288) ([Creswell et al., 2015](#_ENREF_4)). Children on psychotropic medication were included if the dose had been stable for at least one month, and the family agreed to maintain that dose throughout the study. All assessors and coders were independent and blind to treatment group for the duration of the study.

*Overcoming.* Children were treated with a parent-guided self-help CBT program (trial registration: ISRCTN92977593), comprised of the same primary components as the Cool Kids Program ([Lyneham et al., 2003](#_ENREF_11); [Thirlwall et al., 2013](#_ENREF_23)). This consisted of 2–4 in-person sessions and 2–4 telephone sessions. Children on psychotropic medication were included if the dose had been stable for at least one month, and the family agreed to maintain that dose throughout the study. Post treatment assessments were carried out by independent assessors blind to the treatment condition.

A subset of this group with a primary anxiety disorder diagnosis of social phobia also received targeted cognitive bias modification training ([CBM-I; Vassilopoulos, Banerjee, & Prantzalou, 2009](#_ENREF_24)). This trial was not registered. Additionally, participants with highly anxious parents (screened using the Depression Anxiety Stress Scales [DASS] or by meeting Anxiety Disorders Interview Schedule [ADIS] criteria) were randomized to groups in a trial including additional sessions for the parents, which focused on strategies for tolerating children’s negative emotions (trial registration: ISRCTN77196667) ([Hiller et al., 2016](#_ENREF_6)). Children on psychotropic medication were included if the dose had been stable for at least two months, and the family agreed to maintain that dose throughout the study. Assessments were carried out by trained diagnosticians and inter-rater reliability estimates were obtained.

In Oxford, participants came from a basic program delivered by primary health workers as part of a feasibility study ([Creswell et al., 2010; no trial registration](#_ENREF_5)) in addition to an RCT (trial registration: ISRCTN07627865). Children on psychotropic medication were included if the dose had been stable for at least two months, and the family agreed to maintain that dose throughout the study. Post-treatment assessments were carried out by an independent assessor, blind to treatment group and trained to a high level of reliability in the use of the measure.

**Aarhus, Denmark** (n = 124). Participants aged 7-17 years were recruited from the Department of Psychology and Behavioral Sciences, Aarhus University, and all anxiety disorder diagnoses were included. Participants received CBT using the Cool Kids manual ([Lyneham et al., 2003](#_ENREF_11); [Rapee et al., 2006c](#_ENREF_18)). Participants came from two groups: one aged 7–17, from a trial including treatment and waitlist conditions; and another group aged 7–12 from a trial comparing efficacy of traditional group-based treatment with Cool Kids versus a guided self-help version with clinician support (bibliotherapy). The study design of the trials were not preregistered. In both trials, only participants that received in-person CBT were included. Therapists from these trials included a mix of novice and experienced therapists. Treatments were coded as active parental involvement with high CM/TC. Participants were encouraged not to engage in other forms of treatment or change psychopharmacological medication during the treatment. At the site assessments were carried out by blind assessors that were not independent (i.e., they had been part of the study).

**Bergen, Norway** (n = 119). Participants aged 8-13 were recruited from the child part of the “Assessment and Treatment – Anxiety in Children and Adults” study, Haukeland University Hospital, Bergen. Patients referred to outpatient mental health clinics in Western Norway, with a primary diagnosis of separation anxiety, social phobia, or generalized anxiety, received group or individual treatment with the FRIENDS program - 4^th^ edition ([Barrett, 2004](#_ENREF_1); [Barrett, Farrell, Ollendick, & Dadds, 2006](#_ENREF_2)) in a randomized controlled trial (trial registration: NCT00586586) comparing active treatment with a waitlist condition ([Wergeland et al., 2014](#_ENREF_25)). Therapists from these trials included a mix of novice and experienced therapists. All therapists were experienced in working with children. Treatments were coded as having low parental involvement. Youth on psychotropic medication were included if the dosage had been stable for at least 3 months prior study entry and it was kept stable during the treatment (n= 8-13-year-old children). ADIS assessments were carried out by experienced clinicians from participating clinics (not the child’s therapist). Blinding was not possible as assessors worked in the same clinics where treatment was offered.

**Bochum, Germany** (n = 57). Participants aged 5–18, not taking psychotropic medication, were recruited from the Research and Treatment Centre for Mental Health, Ruhr-Universität Bochum (trial registration NCT02077205). Participants received either exposure-based CBT (8–25 sessions, with sessions occurring at least every 2 weeks (coded as low parental involvement, the Coping Cat program ([Kendall, 1994](#_ENREF_10))) coded as low parental involvement, or a family-based version of CBT specifically designed to target separation anxiety disorder coded as active parental involvement with high CM/TC (TAFF; [Schneider et al., 2013](#_ENREF_20); [Schneider & Lavallee, 2013](#_ENREF_21)). Diagnoses were provided separately for parent and child report. The primary diagnosis was selected as being the most severe from either reporter. If the most severe disorder reported by each was of equal severity but was a different diagnosis, the parent-reported diagnosis was selected. Therapists from this site included a mix of novice and experienced therapists. All therapists had a master’s degree in Clinical Psychology and were either in the advanced stages of child CBT training or they were licensed child CBT therapists. Assessments were administered by masters’ level clinicians blind to the treatment condition.

**Basel, Switzerland** (n = 49). Participants aged 5–13 (all with a primary diagnosis of separation anxiety disorder, not taking psychotropic medication) were recruited from the faculty of psychology, University of Basel ([Schneider et al., 2011](#_ENREF_19)). All participants took part in a randomized controlled trial (trial registration: NCT00255112) comparing a family-based version of CBT specifically designed to target separation anxiety disorder coded as active parental involvement with high CM/TC (TAFF; [Schneider et al., 2013](#_ENREF_20); [Schneider & Lavallee, 2013](#_ENREF_21))) with Coping Cat coded as low parental involvement ([Kendall, 1994](#_ENREF_10)). All participants received 16 sessions over 12 weeks. Therapists from this site included a mix of novice and experienced therapists. All therapists had a master’s degree in Clinical Psychology and were either in the advanced stages of child CBT training or were licensed child CBT therapists. Interviews were conducted by trained clinical psychologists or advanced masters students, blinded to group status at all evaluations.

**Groningen, the Netherlands** (n = 37). Participants aged 8 to 17 were recruited from the Department of Child and Adolescent Psychiatry, University of Groningen. The patients were recruited in the context of a larger trial that was preregistered in the Dutch Trial register with number 704 (<http://www.trialregister.nl/trialreg/admin/rctview.asp?TC=704>) ([Hogendoorn et al., 2014](#_ENREF_7)). Only the families that were recruited in Groningen (rather than Amsterdam) were asked to participate in the collaborative Genes for Treatment study. All participants were treated within a randomized controlled trial of Coping Cat (Dutch version) ([Nauta & Scholing, 1998](#_ENREF_15)) including 12 individual child sessions and 2 parent sessions. Treatment was coded as low parental involvement. Therapists conducting the treatment were a mix of novice and experienced therapists. The use of an SSRI was an exclusion criterion for the study, while the use of other medication including e.g. methylphenidate was allowed if kept constant during treatment. Assessments were not-blinded. All assessments were carried out by experienced and trained clinicians that were not the child’s therapist.

**Florida, USA** (n = 50). Participants aged 7–16 (including all primary anxiety disorder diagnoses except posttraumatic stress disorder [PTSD]) were recruited from the Child Anxiety and Phobia Program, Florida International University, Miami to participate in a clinical trial (trial registration: NCT00620958). All participants received 12–14 hour-long sessions of individual manualized CBT. Additionally, two conditions included parental involvement focusing on different parent skills (Relationship Skills Training coded as active parental involvement with low CM/TC or Reinforcement Skills Training coded as active parental involvement with high contingency management/transfer of control). Therapists from these trials included a mix of novice and experienced therapists. Participants on psychiatric medication treatment were included in this trial if they were on a stable dose of medication (i.e., four to eight weeks on a stable dose prior to initial assessment) and maintained the dose during the course of treatment. All post-treatment assessments were delivered by assessors blind to treatment condition.

**Cambridge, UK** (n = 12). Participants aged 8–17 were recruited from the Medical Research Council Cognition and Brain Sciences Unit, Cambridge, UK. Participants were taking part in the Acute Stress Programme for Children and Teenagers (ASPECTS) trial ([Meiser-Stedman et al., 2017](#_ENREF_14)), which recruited individuals exposed to a recent (i.e. in the previous six months) traumatic stressor (i.e. any event that involve the threat of death, severe injury, or threat to bodily integrity, or witnessing such an event). The ASPECTS trial was approved by the UK National Research Ethics Service, Cambridgeshire 1 Research Ethics Committee (10/H0304/11), and registered with the ISRCTN Registry (ISRCTN38352118). Those cases that developed PTSD were randomized to a 10-week waitlist or individual PTSD-specific CBT ([Smith et al., 2007](#_ENREF_22)) which consisted of up to 10 sessions over a 10-week period. Only participants that received treatment were included. Treatment was coded as active parental involvement with low CM/TC. The two therapists delivering treatment were experienced. A change in psychoactive medication in the three months prior to trial baseline assessment was an exclusion criterion. No participants took psychoactive medication during the treatment phase. All post–treatment assessments were carried out by blind, independent assessors.

**Amsterdam, the Netherlands** (n = 4). Participants aged 10–14 were recruited from the Academic Treatment Centre for Parent and Child, University of Amsterdam (UvA) Minds and received either 12 weeks of CBT in individual sessions or 8 weeks of CBT in group sessions, according to the Dutch protocol “Discussing + Doing = Daring.” Treatment was coded as low parental involvement. Diagnoses were provided separately for parent and child report, with the primary diagnosis selected from these data by the trial manager. Therapists in this trial had several years of experience. Participants were not part of a registered trial, and the policy was that if patients were on medication, medication was kept constant during the trial and follow-up period.

|  | GAD | | | SoAD | | | SP | | | SAD | | | Total | | |
| --- | --- | --- | --- | --- | --- | --- | --- | --- | --- | --- | --- | --- | --- | --- | --- |
|  | Ind | Group | Parent | Ind | Group | Parent | Ind | Group | Parent | Ind | Group | Parent | Ind | Group | Parent |
| Aarhus % (n) | 0 | 7.2 (24) | 0 | 0 | 6.3 (9) | 0 | 0 | 25 (15) | 0 | 0 | 29 (34) | 0 | 0 | 13 (82) | 0 |
| Amsterdam | 0 | 0 | 0 | 0 | 0 | 0 | 0 | 2 (1) | 0 | 0 | 1 (1) | 0 | 0 | .3 (2) | 0 |
| Basel | 0 | 0 | 0 | 0 | 0 | 0 | 0 | 0 | 0 | 36 (48) | 0 | 0 | 13 (48) | 0 | 0 |
| Bergen | 9 | 2.4 (8) | 0 | 23 (19) | 9.2 (13) | 0 | 0 | 0 | 0 | 14 (19) | 14 (16) | 0 | 13 (47) | 7.0 (37) | 0 |
| Bochum | 1.2 (1) | 0 | 0 | 13 (11) | 0 | 0 | 30 (14) | 0 | 0 | 9.0 (12) | 0 | 0 | 11 (38) | 0 | 0 |
| Groningen | 5.1 (4) | 0 | 0 | 9.6 (8) | 0 | 0 | 8.7 (4) | 0 | 0 | 3.7 (5) | 0 | 0 | 5.8 (21) | 0 | 0 |
| Florida | 14 (11) | 0 | 0 | 14 (12) | 0 | 0 | 13 (6) | 0 | 0 | 8.2 (11) | 0 | 0 | 11 (40) | 0 | 0 |
| Oxford | 0 | 0 | 3.1 (3) | 0 | 0 | 12 (7) | 0 | 0 | 1 (1) | 0 | 0 | 13 (9) | 5.5 (20) | 0 | 0 |
| Reading | 1.2 (43) | 0 | 58 (56) | 34 (28) | 0 | 60 (35) | 43 (20) | 0 | 92 (31) | 28 (38) | 0 | 72 (48) | 36 (129) | 0 | 71 (170) |
| Sydney | 1.3 (10) | 90 (301) | 39 (38) | 6.0 (5) | 85 (120) | 28 (16) | 4.3 (2) | 73 (43) | 6.7 (6) | 1 (1) | 70 (67) | 15 (10) | 4.9 (18) | 81 (531) | 29 (70) |
| Total | 100 (78) | 100 (333) | 100 (97) | 100 (83) | 100  (142) | 100 (58) | 100 (46) | 100 (59) | 100 (89) | 100 (134) | 100 (118) | 100 (67) | 100 (361) | 100 (652) | 100 (240) |

**Supplementary Table S1.** Percentage of cases per site for treatment formats within diagnostic categories.

Note. GAD=Generalised Anxiety Disorder; SoAD=Social Anxiety Disorder; SP=Specific Phobia; SAD=Separation Anxiety Disorder; Ind= Individual Cognitive Behaviour Therapy; Group= Group Cognitive Behaviour Therapy; Parent= Guided Parent-led Cognitive Behaviour Therapy.

| Generalized Anxiety Disorder | Social Anxiety Disorder |
| --- | --- |
|  |  |
| Separation Anxiety Disorder |  |
|  |  |
| **Supplementary Figure S1.** Mean clinician severity rating (CSR) scores over time across individual CBT, group CBT and parent-led CBT for children with GAD, SAD, SoAD. | |

**Supplementary Table S2.** Results of linear and logistic mixed models examining the relationship between primary diagnosis and treatment format in the full sample using all follow-up points.

|  | *Response* | | *Remission* | |
| --- | --- | --- | --- | --- |
|  | β (S.E.) | *95% CI* | OR (S.E.) | *95% CI* |
| Time | -.28* (.04) | [-.37, -.19] | 3.2* (.74) | [2.0, 5.0] |
| Severity of primary diagnosis at baseline^1^ | .18* (.02) | [.13, .22] | .51* (.06) | [.41, .64] |
| CBT Treatment |  |  |  |  |
| Individual | ^b^ | ^b^ | ^b^ | ^b^ |
| Group-based^2^ | .17 (.11) | [.-.04, .38] | .44 (.22) | [.16, 1.1] |
| Guided parent-led^2^ | .01 (.14) | [-.27, .29] | .28 (.19) | [.07, 1.1] |
| Diagnosis |  |  |  |  |
| SoAD | ^b^ | ^b^ | ^b^ | ^b^ |
| GAD | -.40* (.05) | [-.50, -.30] | 5.2* (1.3) | [3.1, 8.5] |
| SP | -.24* (.07) | [-.37, -.07] | 2.7* (.93) | [1.4, 5.3] |
| SAD | -.29* (.06) | [-.41, -.18] | 3.6* (1.0) | [2.1, 6.3] |
| Age | .00 (.01) | [-.02, .03] | 1.0* (.06) | [.90, 1.1] |
| Gender | .09 (.04) | [.02, .17] | .75 (.14) | [.52, 1.1] |

^1^ Standardized regression coefficients (β) significantly different than zero indicate association with symptom severity after treatment. Odds ratios predicting a higher likelihood of remission are significantly greater than one, whereas variables predicting a lower likelihood of remission have odds ratios of significantly less than 1.

^2^ Standardized regression coefficients (β) significantly different than zero indicate higher (negative value) or lower (positive value) changes in symptom severity compared to the reference category. Odds ratios predicting a higher likelihood of remission relative to the reference category are significantly greater than one, whereas variables predicting a lower likelihood of remission relative to the reference category have odds ratios of significantly less than 1.

^b^ Represents the reference category.

Note. GAD=Generalised Anxiety Disorder; SoAD=Social Anxiety Disorder; SP=Specific Phobia; SAD=Separation Anxiety Disorder; CBT=Cognitive Behaviour Therapy. ^*^*p*<.006

**Supplementary Table S3.** Means (standard errors) of the relationship between primary diagnosis and treatment format in severity scores (CSR) at pre, post, and follow-up.

|  | *pre* | *post* | *follow-up* |  | *pre* | *post* | *follow-up* |
| --- | --- | --- | --- | --- | --- | --- | --- |
| GAD |  |  |  | SP |  |  |  |
| Individual CBT | 6.20 (.04) | 2.91 (.37) | 1.71 (.33) | Individual CBT | 6.28 (.17) | 2 (.37) | 1.34 (.36) |
| Group CBT | 6.41 (.05) | 2.69 (.28) | 2.51 (.22) | Group CBT | 6.75 (.13) | 3.83 (.35) | 2.69 (.31) |
| Parent led CBT | 5.58 (.08) | 1.96 (.32) | 1.73 (.37) | Parent led CBT | 5.84 (.14) | 3.93 (.42) | 2.14 (.46) |
|  |  |  |  |  |  |  |  |
| SoAD |  |  |  | SAD |  |  |  |
| Individual CBT | 6.14 (.06) | 3.53 (.32) | 3 (.4) | Individual CBT | 6.25 (.05) | 2.47 (.35) | 2.25 (.25) |
| Group CBT | 6.37 (.08) | 3.79 (.35) | 3.46 (.41) | Group CBT | 6.68 (.09) | 3.14 (.38) | 2.64 (.23) |
| Parent led CBT | 5.5 (.12) | 3.35 (.36) | 1.78 (.63) | Parent led CBT | 5.63 (.10) | 3.34 (.44) | 1.87 (.41) |

Note. GAD=Generalised Anxiety Disorder; SoAD=Social Anxiety Disorder; SP=Specific Phobia; SAD=Separation Anxiety Disorder; CBT=Cognitive Behaviour Therapy.

**Supplementary Table S4a.** Results of linear mixed models examining the relationship between primary diagnosis and treatment format on severity scores (CSR) using the post time point only.

|  | *GAD* | | *SoAD* | | *SP* | | *SAD* | |
| --- | --- | --- | --- | --- | --- | --- | --- | --- |
|  | β (S.E.) | *95% CI* | β (S.E.) | *95% CI* | β (S.E.) | *95% CI* | β (S.E.) | *95% CI* |
| Severity of primary diagnosis at baseline^1^ | .13* (.04) | [.05, .20] | .28* (.05) | [.19, .37] | .05 (.07) | [-.09, .20] | .24* (.05) | [.13, .34] |
| CBT Treatment |  |  |  |  |  |  |  |  |
| Individual | ^b^ | ^b^ | ^b^ | ^b^ | ^b^ | ^b^ | ^b^ | ^b^ |
| Group-based^2^ | -.09 (.18) | [-.43, 26] | .10 (.17) | [-.23, .43] | .73* (.21) | [.33, 1.1] | .26 (.18) | [-.09, .62] |
| Guided parent-led^2^ | -.37 (.20) | [-.76, .01] | -.08 (.20) | [-.45, .31] | .78* (.22) | [.34, 1.2] | .34 (.23) | [-.10, .78] |
| Age | .01 (.02) | [-.03, .05] | -.00 (.03) | [-.06, .06] | .05 (.04) | [-.04, .14] | .01 (.03) | [-.05, .07] |
| Gender | .13 (.07) | [-.00, .26] | .05 (.09) | [-.12, .22] | .02 (.15) | [-.26, .31] | .10 (.10) | [-.08, .29] |

Note. GAD=Generalised Anxiety Disorder; SoAD=Social Anxiety Disorder; SP=Specific Phobia; SAD=Separation Anxiety Disorder; CBT=Cognitive Behaviour Therapy.

^1^ Standardized regression coefficients (β) significantly different than zero indicate association with symptom severity after treatment.

^2^ Standardized regression coefficients (β) significantly different than zero indicate higher (negative value) or lower (positive value) changes in symptom severity compared to the reference category.

^b^ Reference category

^*^*p*<.006

**Supplementary Table S4b.** Results of logistic mixed models examining the relationship between primary diagnosis and treatment format on severity scores (CSR) using the post time point only.

|  | *GAD* | | *SoAD* | | *SP* | | *SAD* | |
| --- | --- | --- | --- | --- | --- | --- | --- | --- |
|  | *OR* (S.E.) | *95% CI* | *OR* (S.E.) | *95% CI* | *OR* (S.E.) | *95% CI* | *OR* (S.E.) | *95% CI* |
| Severity of primary diagnosis at baseline^1^ | .68 (.13) | [.46, .99] | .57* (.08) | [.43, .76] | .81 (.16) | [.54, 1.2] | .60* (.09) | [.44, .82] |
| CBT Treatment |  |  |  |  |  |  |  |  |
| Individual | ^b^ | ^b^ | ^b^ | ^b^ | ^b^ | ^b^ | ^b^ | ^b^ |
| Group-based^2^ | 1.3 (.37) | [.76, 2.3] | .72 (.39) | [.25, 2.1] | .23 (.16) | [.05, .92] | .44 (.22) | [.15, 1.2] |
| Guided parent-led^2^ | 1.7 (.58) | [.87, 3.3] | .68 (.42) | [.20, 2.3] | .14* (.10) | [.06, .92] | .19 (.12) | [.05, .67] |
| Age | 1.0 (.06) | [.92, 1.2] | .95 (.09) | [.79, 1.1] | .85 (.11) | [.67, 1.1] | .99 (.08) | [.83, 1.2] |
| Gender | .67 (.13) | [.46, .98] | 1.2 (.32) | [.70, 2.0] | 1.3 (.51) | [.64, 2.8] | .57 (.15) | [.33, .96] |

Note. GAD=Generalised Anxiety Disorder; SoAD=Social Anxiety Disorder; SP=Specific Phobia; SAD=Separation Anxiety Disorder; CBT=Cognitive Behaviour Therapy.

^1^ Odds ratios predicting a higher likelihood of remission are significantly greater than one, whereas variables predicting a lower likelihood of remission have odds ratios of significantly less than 1.

^2^ Odds ratios predicting a higher likelihood of remission relative to the reference category are significantly greater than one, whereas variables predicting a lower likelihood of remission relative to the reference category have odds ratios of significantly less than 1.

^b^ Reference category.

p < .006*

**Supplementary Table S5a.** Results of linear mixed models examining the relationship between primary diagnosis and treatment format on severity scores (CSR) using the follow-up time point only.

|  | *GAD* | | *SoAD* | | *SP* | | *SAD* | |
| --- | --- | --- | --- | --- | --- | --- | --- | --- |
|  | *β* (S.E.) | *95% CI* | *β* (S.E.) | *95% CI* | *β* (S.E.) | *95% CI* | *β* (S.E.) | *95% CI* |
| Severity of primary diagnosis at baseline^1^ | .10 (.04) | [.02, .17] | .21* (.05) | [.10, .31] | .10 (.15) | [.48, 1.1] | .20* (.06) | [.08, .32] |
| CBT Treatment |  |  |  |  |  |  |  |  |
| Individual | ^b^ | ^b^ | ^b^ | ^b^ | ^b^ | ^b^ | ^b^ | ^b^ |
| Group-based^2^ | .32 (.15) | [.01, .63] | .18 (.1820 | [-.21, .58] | .54 (.20) | [.15, .94] | .15 (.14) | [-.12, .42] |
| Guided parent-led^2^ | .01 (.20) | [.-.38, .40] | -.48 (.30) | [-1.1, .11] | .32 (.24) | [-.14, .78] | -.15 (.19) | [-.53, .22] |
| Age | -.04 (.02) | [-.08, -.00] | .01 (.03) | [-.04, .08] | .03 (.05) | [-.07, .13] | -.03 (.04) | [-.10, .05] |
| Gender | .07 (.07) | [-.07, .21] | .24 (.10) | [.04, .43] | -.09 (.15) | [-.40, .22] | .20 (.12) | [-.04, .43] |

Note. GAD=Generalised Anxiety Disorder; SoAD=Social Anxiety Disorder; SP=Specific Phobia; SAD=Separation Anxiety Disorder; CBT=Cognitive Behaviour Therapy.

^1^ Standardized regression coefficients (β) significantly different than zero indicate association with symptom severity after treatment.

^2^ Standardized regression coefficients (β) significantly different than zero indicate higher (negative value) or lower (positive value) changes in symptom severity compared to the reference category.

^b^ Reference category

^*^*p*<.006

**Supplementary Table S5b.** Results of logistic mixed models examining the relationship between primary diagnosis and treatment format on severity scores (CSR) using the follow-up time point only.

|  | *GAD* | | *SoAD* | | *SP* | | *SAD* | |
| --- | --- | --- | --- | --- | --- | --- | --- | --- |
|  | *OR* (S.E.) | *95% CI* | *OR* (S.E.) | *95% CI* | *OR* (S.E.) | *95% CI* | *OR* (S.E.) | *95% CI* |
| Severity of primary diagnosis at baseline^1^ | .67 (.21) | [.36, 1.2] | .47 (.16) | [.24, .91] | .51 (.27) | [.18, 1.4] | .33* (.13) | [.15, .75] |
| CBT Treatment |  |  |  |  |  |  |  |  |
| Individual | ^b^ | ^b^ | ^b^ | ^b^ | ^b^ | ^b^ | ^b^ | ^b^ |
| Group-based^2^ | .22 (.35) | [.01, 5.3] | .53 (.46) | [.11, 2.5] | .16 (.22) | [.01, 2.3] | 1.3 (1.0) | [.28, 6.0] |
| Guided parent-led^2^ | .92 (2.2) | [.01, 93] | 7.8 (12) | [.37, 158] | .07 (.12) | [.00, 1.9] | 1.1 (1.2) | [.12, 9.0] |
| Age | 1.1 (.17) | [.86, 1.5] | .90 (.15) | [.71, 1.1] | 1.1 (.34) | [.64, 2.0] | 1.2 (.24) | [.77, 1.7] |
| Gender | .78 (.38) | [.30, 2.0] | .51 (.28) | [.17, 1.5] | 2.1 (2.0) | [.33, 14] | .54 (.35) | [.15, 1.9] |

Note. GAD=Generalised Anxiety Disorder; SoAD=Social Anxiety Disorder; SP=Specific Phobia; SAD=Separation Anxiety Disorder; CBT=Cognitive Behaviour Therapy.

^1^ Odds ratios predicting a higher likelihood of remission are significantly greater than one, whereas variables predicting a lower likelihood of remission have odds ratios of significantly less than 1.

^2^ Odds ratios predicting a higher likelihood of remission relative to the reference category are significantly greater than one, whereas variables predicting a lower likelihood of remission relative to the reference category have odds ratios of significantly less than 1.

^b^ Reference category.

p < .006*

**References**

Barrett, P. M. (2004). *FRIENDS for Life program - Group leader's workbook for children* (4th ed. ed.). Brisbane, Queensland: Australian Academic Press.

Barrett, P. M., Farrell, L. J., Ollendick, T. H., & Dadds, M. (2006). Long-Term Outcomes of an Australian Universal Prevention Trial of Anxiety and Depression Symptoms in Children and Youth: An Evaluation of the Friends Program. *Journal of Clinical Child & Adolescent Psychology, 35*, 403-411. doi: 10.1207/s15374424jccp3503_5

Creswell, C., Cruddace, S., Gerry, S., E., M., J., M., Murray, L., . . . Cooper, P. J. (2007). Treatment of childhood anxiety disorder in the context of maternal anxiety disorder: A randomised controlled trial *Health Technology Assessment*

Creswell, C., Cruddace, S., Gerry, S., Gitau, R., McIntosh, E., Mollison, J., . . . Cooper, P. J. (2015). Treatment of childhood anxiety disorder in the context of maternal anxiety disorder: a randomised controlled trial and economic analysis. *Health technology assessment (Winchester, England), 19*, 1-184, vii-viii. doi: 10.3310/hta19380

Creswell, C., Hentges, F., Parkinson, M., Sheffield, P., Willetts, L., & Cooper, P. (2010). Feasibility of guided cognitive behaviour therapy (CBT) self-help for childhood anxiety disorders in primary care. *Mental health in family medicine, 7*, 49-57.

Hiller, R. M., Apetroaia, A., Clarke, K., Hughes, Z., Orchard, F., Parkinson, M., & Creswell, C. (2016). The effect of targeting tolerance of children's negative emotions among anxious parents of children with anxiety disorders: A pilot randomised controlled trial. *Journal of Anxiety Disorders, 42*, 52-59. doi: 10.1016/j.janxdis.2016.05.009

Hogendoorn, S. M., Prins, P. J. M., Boer, F., Vervoort, L., Wolters, L. H., Moorlag, H., . . . de Haan, E. (2014). Mediators of cognitive behavioral therapy for anxiety-disordered children and adolescents: cognition, perceived control, and coping. *Journal of clinical child and adolescent psychology : the official journal for the Society of Clinical Child and Adolescent Psychology, American Psychological Association, Division 53, 43*, 486-500. doi: 10.1080/15374416.2013.807736

Hudson, J. L., Newall, C., Rapee, R. M., Lyneham, H. J., Schniering, C. C., Wuthrich, V. M., . . . Gar, N. S. (2013). The Impact of Brief Parental Anxiety Management on Child Anxiety Treatment Outcomes: A Controlled Trial. *Journal of Clinical Child & Adolescent Psychology*, 1-11. doi: 10.1080/15374416.2013.807734

Hudson, J. L., Rapee, R. M., Deveney, C., Schniering, C. A., Lyneham, H. J., & Bovopoulos, N. (2009). Cognitive-Behavioral Treatment Versus an Active Control for Children and Adolescents With Anxiety Disorders: A Randomized Trial. *Journal of the American Academy of Child & Adolescent Psychiatry, 48*, 533-544. doi: <http://dx.doi.org/10.1097/CHI.0b013e31819c2401>

Kendall, P. C. (1994). Treating anxiety disorders in children: Results of a randomized clinical trial. *Journal of Consulting and Clinical Psychology, 62*, 100-110. doi: 10.1037/0022-006X.62.1.100

Lyneham, H. J., Abbott, M. J., Wignall, A., & Rapee, R. M. (2003). *The Cool Kids Anxiety Treatment Programme*. Sydney: MUARU, Macquarie Unversity.

Lyneham, H. J., & Rapee, R. M. (2006). Evaluation of therapist-supported parent-implemented CBT for anxiety disorders in rural children. *Behaviour Research and Therapy, 44*, 1287-1300. doi: <http://dx.doi.org/10.1016/j.brat.2005.09.009>

Manassis, K., Lee, T. C., Bennett, K., Zhao, X. Y., Mendlowitz, S., Duda, S., . . . Wood, J. J. (2014). Types of Parental Involvement in CBT With Anxious Youth: A Preliminary Meta-Analysis. *Journal of Consulting and Clinical Psychology, 82*, 1163-1172. doi: 10.1037/a0036969

Meiser-Stedman, R., Smith, P., McKinnon, A., Dixon, C., Trickey, D., Ehlers, A., . . . Dalgleish, T. (2017). Cognitive therapy as an early treatment for post-traumatic stress disorder in children and adolescents: a randomized controlled trial addressing preliminary efficacy and mechanisms of action. *Journal of child psychology and psychiatry, and allied disciplines, 58*, 623-633. doi: 10.1111/jcpp.12673

Nauta, M. H., & Scholing, A. (1998). *Cognitieve gedragstherapie bij kinderen en jongeren met angststoornissen: een protocol van 12 sessies. Handleiding voor de therapeut.* Groningen: Rijksuniversiteit Groningen (Klinische en Ontwikkelingspsychologie).

Rapee, R., Lyneham, H., Schniering, C., Wuthrich, V., Abbott, M., Hudson, J., & Wignall, A. (2006a). *The Cool Kids® Child and Adolescent Anxiety Program*. Sydney: Centre for Emotional Health, Macquarie University.

Rapee, R. M., Abbott, M. J., & Lyneham, H. J. (2006b). Bibliotherapy for children with anxiety disorders using written materials for parents: A randomized controlled trial. *Journal of Consulting and Clinical Psychology, 74*, 436-444. doi: 10.1037/0022-006x.74.3.436

Rapee, R. M., Lyneham, H. J., Schniering, C. A., Wuthrich, V. M., Abbott, M. J., Hudson, J. L., & Wignall, A. (2006c). *Cool Kids "Chilled" Adolescent Anxiety Program*. Sydney: MUARU, Macquarie University.

Schneider, S., Blatter-Meunier, J., Herren, C., Adornetto, C., In-Albon, T., & Lavallee, K. (2011). Disorder-specific cognitive-behavioral therapy for separation anxiety disorder in young children: a randomized waiting-list-controlled trial. *Psychotherapy and Psychosomatics, 80*, 206-215. doi: 10.1159/000323444

Schneider, S., Blatter-Meunier, J., Herren, C., In-Albon, T., Adornetto, C., Meyer, A., & Lavallee, K. L. (2013). The efficacy of a family-based cognitive-behavioral treatment for separation anxiety disorder in children aged 8–13: A randomized comparison with a general anxiety program. *Journal of Consulting and Clinical Psychology, 81*, 932-940. doi: 10.1037/a0032678

Schneider, S., & Lavallee, K. (2013). Separation Anxiety Disorder. In E. C.A. & O. T. (Eds.), *The Wiley-Blackwell Handbook of The Treatment of Childhood and Adolescent Anxiety* (pp. 301-334): Wiley-Blackwell.

Smith, P., Yule, W., Perrin, S., Tranah, T., Dalgleish, T. I. M., & Clark, D. M. (2007). Cognitive-Behavioral Therapy for PTSD in Children and Adolescents: A Preliminary Randomized Controlled Trial. *Journal of the American Academy of Child & Adolescent Psychiatry, 46*, 1051-1061. doi: <http://dx.doi.org/10.1097/CHI.0b013e318067e288>

Thirlwall, K., Cooper, P. J., Karalus, J., Voysey, M., Willetts, L., & Creswell, C. (2013). Treatment of child anxiety disorders via guided parent-delivered cognitive-behavioural therapy: randomised controlled trial. *The British Journal of Psychiatry, 203*, 436-444. doi: 10.1192/bjp.bp.113.126698

Vassilopoulos, S. P., Banerjee, R., & Prantzalou, C. (2009). Experimental modification of interpretation bias in socially anxious children: Changes in interpretation, anticipated interpersonal anxiety, and social anxiety symptoms. *Behaviour Research and Therapy, 47*, 1085-1089. doi: <http://dx.doi.org/10.1016/j.brat.2009.07.018>

Wergeland, G. J. H., Fjermestad, K. W., Marin, C. E., Haugland, B. S.-M., Bjaastad, J. F., Oeding, K., . . . Heiervang, E. R. (2014). An effectiveness study of individual vs. group cognitive behavioral therapy for anxiety disorders in youth. *Behaviour Research and Therapy, 57*, 1-12. doi: <http://dx.doi.org/10.1016/j.brat.2014.03.007>
